# High Seroprevalence of Helicobacter Pylori Infection in Inmates: A Case Control Study in a Northern Mexican City

**DOI:** 10.4021/gr583w

**Published:** 2014-01-15

**Authors:** Cosme Alvarado-Esquivel, Jesus Hernandez-Tinoco, Luis Francisco Sanchez-Anguiano, Agar Ramos-Nevarez, Sandra Margarita Cerrillo-Soto, Leandro Saenz-Soto

**Affiliations:** a Biomedical Research Laboratory, Faculty of Medicine and Nutrition, Juarez University of Durango State, Avenida Universidad S/N, 34000 Durango, Mexico; b Institute for Scientific Research, Juarez University of Durango State, Avenida Universidad S/N, 34000 Durango, Mexico; c Clinica de Medicina Familiar, Instituto de Seguridad y Servicios Sociales de los Trabajadores del Estado, Predio Canoas S/N, 34079 Durango, Mexico

**Keywords:** *Helicobacter pylori*, Seroprevalence, Inmates, Epidemiology, Mexico

## Abstract

**Background:**

The epidemiology of *Helicobacter pylori* infection in inmates has not been previously studied. Therefore, we determine the seroepidemiology of *H. pylori* infection in inmates.

**Methods:**

Through a case-control study, inmates from a state correctional facility in Durango, Mexico and subjects without incarceration of the same city were examined for the presence of anti-*H. pylori* IgG antibodies using enzyme-linked immunoassays. Seroprevalence association with socio-demographic, incarceration, clinical and behavioral characteristics of the inmates was also investigated.

**Results:**

Antibodies to *H. pylori* were found in 140 (83.3%) of 168 inmates and in 101 (60.1%) of 168 controls. Seroprevalence of anti-*H. pylori* IgG antibodies was significantly higher in inmates than in controls (OR = 3.32; 95% CI: 1.93 - 5.71; P = 0.000002). The seroprevalence of *H. pylori* infection was not influenced by gender, age, or socioeconomic status of inmates. Seropositivity to *H. pylori* was found in 3 of 3 inmates with peptic ulcer and in 1 of 2 inmates with gastritis. The seroprevalence of *H. pylori* exposure was high regardless the jail section, duration (years) in incarceration and number of incarcerations. Multivariate analysis revealed that *H. pylori* exposure was positively associated with having tattoos (OR = 3.34; 95% CI: 1.14 - 9.70; P = 0.02), and negatively associated with drug abuse (OR = 0.28; 95% CI: 0.11 - 0.70; P = 0.007).

**Conclusions:**

Seroprevalence of *H. pylori* exposure in inmates is higher than those found in non-incarcerated people and other populations in the region. Results indicate that inmates may represent a new risk group for *H. pylori* exposure. Results warrant for further research on the potential role of incarceration and behavioral features of inmates for *H. pylori* infection.

## Introduction

Infections with the bacterium *Helicobacter pylori* are common among the world’s population [1]. The clinical spectrum of infections with *H. pylori* varies from asymptomatic state to severe gastric disease. Infections with *H. pylori* cause chronic gastritis, peptic ulcer, gastric mucosa-associated lymphoid tissue lymphoma and gastric cancer [1-4]. In addition, *H. pylori* infections have been linked with extra-gastric diseases including sideropenic anemia, idiopathic thrombocytopenic purpura and other conditions [4-6]. A number of transmission routes for *H. pylori* have been reported including oral-oral or fecal-oral [7], person-to-person [8] and consumption of contaminated water [7, 9].

The epidemiology of *H. pylori* infection in inmates has not been studied. Inmates should be considered a group of population with epidemiologic importance for *H. pylori* infection since they have a number of relevant characteristics for acquiring such infection. In Mexico and probably in other countries, inmates live mostly in overcrowding conditions. Overcrowding may facilitate the oral-oral and fecal-oral routes of *H. pylori* infection. In addition, overcrowding and confinement may facilitate the person to person route of infection with *H. pylori* too. Therefore, this study was aimed to determine the seroprevalence of anti-*H. pylori* antibodies in inmates in Durango City, Mexico. In addition, seroprevalence association with socio-demographic, clinical, incarceration and behavioral characteristics in inmates was examined.

## Materials and Methods

### Study design and study populations

Through a case-control study using serum samples from previous *Toxoplasma gondii* and viral hepatitis serosurveys [10, 11], 168 inmates (cases) and 168 controls were examined for the presence of anti-*H. pylori* IgG antibodies. Inclusion criteria for the inmates were: 1) current incarceration in the state correctional in Durango City, Mexico; 2) aged 18 years and older; 3) any gender; 4) incarceration for at least 6 months; and 5) who accepted to participate in the study. Inmates included in the study were 18 - 73 (mean = 33.2 ± 10.79) years old: 129 were males and 39 were females. Controls were subjects without incarceration from the same Durango, City. Controls were matched with cases by age and gender. They were 18 - 73 (mean = 33.7 ± 11.60) years old: 129 were males and 39 were females. Age was comparable between cases and controls (P = 0.73).

### Socio-demographic, clinical, incarceration and behavioral data

A questionnaire was administered to collect socio-demographic, clinical, incarceration and behavioral characteristics of the participants. Socio-demographic items included age, gender, birthplace, residence, marital status, occupation and socioeconomic level. Clinical items included the presence of underlying diseases in general and gastric disease in particular. In women, obstetric history was obtained. Incarceration characteristics assessed included number of incarcerations, jail section and duration of current incarceration. Behavioral items included foreign travel, alcoholism, drug abuse, piercing and tattoos.

### Serologic detection of *H. pylori* antibodies

A commercially available enzyme-linked immunosorbent assay (ELISA) kit, Anti-*H. pylori* IgG AccuBind ELISA (Monobind Inc, Lake Forest, California) was used to detect IgG antibodies against *H. pylori* in the serum of the participants. The ELISA used allows qualitative and quantitative analyses of anti-*H. pylori* IgG antibodies. Anti-*H. pylori* IgG antibody levels were expressed as Units (U)/mL, and a value higher than 20 U/mL was considered a positive result. All tests were performed following the manufacturer’s instructions.

### Statistical analysis

Analyses were performed using the Microsoft Excel 2010, Epi Info version 3.5.4 software (Centers for Disease Control and Prevention: http://wwwn.cdc.gov/epiinfo/) and SPSS version 15.0 software (SPSS Inc., Chicago, Illinois). Age between cases and controls was compared by the Student’s t test. Pearson’s chi-square test was used to determine inmate characteristics associated with *H. pylori* seropositivity. In addition, inmates characteristics with a P value ≤ 0.25 obtained in the bivariate analysis were entered into a multivariate analysis using a conditional backward stepwise logistic regression analysis. Odds ratios (OR) and 95% confidence intervals (CI) were calculated, and a P value < 0.05 was considered statistically significant.

### Ethics statement

Only archival serum samples and data from previous surveys were examined in the present study. However, in the previous surveys, the purpose and procedures of the studies were explained to all participants, and a written informed consent was obtained from each participant. This study was approved by the Ethical Committee of the Instituto de Seguridad y Servicios Sociales de los Trabajadores del Estado in Durango City.

## Results

Anti-*H. pylori* IgG antibodies were detected in 140 (83.3%) of 168 inmates and in 101 (60.1%) of 168 controls. Seroprevalence of anti-*H. pylori* IgG antibodies was significantly higher in inmates than in controls (OR = 3.32; 95% CI: 1.93 - 5.71; P = 0.000002). Stratification by gender also showed differences in seroprevalence between inmates and controls. Anti-*H. pylori* IgG antibodies were detected in 33 (84.6%) of 39 female inmates and in 22 (56.4%) of 39 female controls. Seroprevalence of anti-*H. pylori* IgG antibodies was significantly higher in female inmates than in female controls (OR = 4.25; 95% CI: 1.30 - 14.43; P = 0.006). Anti-*H. pylori* IgG antibodies were detected in 107 (82.9%) of 129 male inmates and in 79 (61.2%) of 129 male controls. Seroprevalence of anti-*H. pylori* IgG antibodies was significantly higher in male inmates than in male controls (OR = 3.08; 95% CI: 1.66 - 5.73; P = 0.0001). Of the 140 *H. pylori* IgG positive inmates, 90 (64.3%) had IgG levels higher than 100 U/mL, 21 (15.0%) between 51 and 100 U/mL, and 29 (20.7%) between 21 and 50 U/mL. Anti-*H. pylori* IgG antibody levels were similar in male and female inmates (P = 0.54).

Of the socio-demographic and incarceration characteristics (Table 1), only two characteristics had P values ≤ 0.25: marital status (P = 0.01) and duration of current incarceration (P = 0.12). Other socio-demographic and incarceration characteristics in inmates including age, gander, birth place, residence, occupation, socioeconomic status, jail section and number of incarcerations had P values > 0.25.

**Table 1:** Socio-Demographic and Incarceration Characteristics of Inmates and Seroprevalence of *H. Pylori* Infection

Characteristic	No. of subjects tested^a^	Prevalence of *H. pylori* infection		P value
		No.	%	
Gender				
Male	129	107	82.9	0.8
Female	39	33	84.6	
Age groups (years)				
30 or less	85	69	81.2	0.54
31 - 50	69	58	84.1	
> 50	14	13	92.9	
Birth place				
Durango State	120	100	83.3	1
Other Mexican state or abroad	48	40	83.3	
Residence				
Durango State	135	113	83.7	0.79
Other Mexican State	33	27	81.8	
Marital status				
Single	44	37	84.1	0.01
Married	72	58	81.0	
Divorced	4	1	25.0	
Living together	41	37	90.2	
Widowed	6	6	100.0	
Occupation				
Laborer^b^	153	127	83.0	0.52
Non-laborer^c^	15	13	86.7	
Socio-economic level				
Low	106	90	84.9	0.33
Medium	52	40	76.9	
High	3	3	100.0	
Jail section				
A	24	21	87.5	0.91
B	3	2	66.7	
C	38	32	84.2	
D	41	34	82.9	
E	62	51	82.3	
Number of incarcerations				
One	130	108	83.1	0.86
Two or more	38	32	84.2	
Duration (years) of current incarceration				
0.5 - 1	47	41	87.2	0.12
1.1 - 2	56	51	91.1	
2.1 - 3	23	17	73.9	
3.1 - 5	26	19	73.1	
More than 5	16	12	75.0	

^a^Subjects with available data; ^b^Laborer: Agriculture, construction worker, business, driver, factory worker, other; ^c^Non-laborer: student or housekeeping.

The seroprevalence of *H. pylori* infection in healthy inmates (82.0%) was comparable with that (85.5%) found in ill inmates (P = 0.57). There were 3 inmates suffering from peptic ulcer and all 3 were positive for anti-*H. pylori* antibodies. In addition, there were 2 inmates suffering from gastritis and one of them was positive for anti-*H. pylori* antibodies. None of the obstetric characteristics in women including pregnancies, deliveries, cesarean sections and abortions was associated with *H. pylori* seropositivity.

Of the behavioral characteristics of inmates examined (Table 2), the following variables had P values ≤ 0.25 in the bivariate analysis: national trips (P = 0.24), traveled abroad (P = 0.22), drug abuse (P = 0.04) and having tattoos (P = 0.20). Multivariate analysis of socio-demographic, incarceration and behavioral characteristics of inmates that had P values ≤ 0.25 in the bivariate analysis revealed that only 2 characteristics were associated with *H. pylori* seropositivity: having tattoos had a positive association (OR = 3.34; 95% CI: 1.14 - 9.70; P = 0.02), and drug abuse had a negative association (OR = 0.28; 95% CI: 0.11 - 0.70; P = 0.007).

**Table 2:** Bivariate Analysis of Selected Behavioral Characteristics in Inmates and *H. Pylori* Seroprevalence

Characteristic	Subjects tested^a^ No.	Prevalence of *H. pylori* infection		P value
		No.	%	
National trips				
Yes	80	64	80.0	0.24
No	83	72	86.7	
Traveled abroad				
Yes	61	48	78.7	0.22
No	107	92	86.0	
Drug abuse				
Yes	67	51	76.1	0.04
No	100	88	88.0	
Alcoholism				
Yes	106	88	83.0	0.88
No	62	52	83.9	
Tattoos				
Yes	53	47	88.7	0.20
No	115	93	80.9	
Piercing				
Yes	60	49	81.7	0.66
No	108	91	84.3	

a Subjects with available data.

## Discussion

In the present study, a statistically significant difference in *H. pylori* seropositivity between inmates and controls was found. Remarkably, inmates had a higher seroprevalence of *H. pylori* exposure than controls. In addition, the seroprevalence found in inmates (83.3%) represents the highest seroprevalence reported in people in the region so far. In recent studies in ethnic groups in Durango, Mexico, the seroprevalences of *H. pylori* exposure in Mennonites [12] and Tepehuanos [13] were 50.7% and 66.0%, respectively. The seroprevalence found in inmates is also higher than a 66.7% seroprevalence found in waste pickers in Durango City [14]. Furthermore, the seroprevalence found in inmates is higher than the mean national seroprevalence (66%) reported in Mexico [15]. It is not clear why inmates had a higher seroprevalence of *H. pylori* exposure than controls. However, all known routes for *H. pylori* infection including oral-oral or fecal-oral [7], person-to-person [8] and consumption of contaminated water [7, 9] might be present in inmates. In contrast, such routes of transmission in non-incarcerated people might be less frequent. Inmates live mostly in overcrowding conditions in Mexico and such factor might account for transmission of *H. pylori* infection by the oral-oral and fecal-oral routes. Crowding has been found associated with *H. pylori* infection [15-17] and is clearly an important factor that could contribute for explaining the high seroprevalence of *H. pylori* in inmates. Of the socio-demographic, incarceration and behavioral characteristics of inmates, multivariate analysis revealed that *H. pylori* seropositivity was positively associated with having tattoos and negatively associated with drug abuse (OR = 0.28; 95% CI: 0.11 - 0.70; P = 0.007). Such associations were unexpected. To the best of our knowledge *H. pylori* seropositivity has not been associated with having tattoos and drug abuse. It is not clear why inmates with tattoos had a higher seroprevalence of *H. pylori* exposure than those without tattoos. It is uncertain whether *H. pylori* can be transmitted by tattooing. It is possible that inmates with tattoos have had an unknown behavioral risk for *H. pylori* infection. Further studies to confirm or challenge the association of *H. pylori* infection with having tattoos are needed. On the other hand, the negative association of drug abuse with *H. pylori* is intriguing too. We are not aware of previous reports of such association. It is not clear why inmates with drug abuse had a lower seroprevalence of *H. pylori* exposure than inmates without drug abuse. This finding might just indicate that drug abuse did not have any role in *H. pylori* infection. Nevertheless, it raises the question whether any drug used by inmates might have an adverse effect against *H. pylori*. It is also possible that inmates without drug abuse have had an unknown behavioral risk factor for *H. pylori*. Further studies to confirm or challenge the negative association of *H. pylori* infection with drug abuse are needed.

The seroprevalence of *H. pylori* exposure in inmates is higher than those found in non-incarcerated people and other reported seroprevalences in the region. Results indicate that inmates represent a new risk group for *H. pylori* exposure. Results warrant for further research on the potential role of incarceration and behavioral features of inmates for *H. pylori* infection.
